# Supplementary material for: High-Sensitivity Ammonia Sensors with Carbon Nanowall Active Material via Laser-Induced Transfer
Source: Nanomaterials (Basel). 2022 Aug 17;12(16):2830. doi: 10.3390/nano12162830 (PMC9413251; doi:10.3390/nano12162830)
Supplement: Supplementary file 1 [file nanomaterials-12-02830-s001.zip › nanomaterials-1836341-supplementary.pdf]

nanomaterials

MDPI

Supplementary Materials

High-Sensitivity Ammonia Sensors with Carbon Nanowall Active Material via Laser-Induced Transfer

Alexandra Palla-Papavlu [1,*], Sorin Vizireanu [1], Mihaela Filipescu [1] and Thomas Lippert [2,3]

[1] Lasers Department, National Institute for Lasers, Plasma, and Radiation Physics, Atomiştilor 409, 077125 Măgurele, Romania
[2] Laboratory of Inorganic Chemistry, Department of Chemistry and Applied Biosciences, ETH Zurich, 8093 Zurich, Switzerland
[3] Laboratory of Multiscale Materials Experiments, Paul Scherrer Institute, 5232 Villigen, Switzerland
* Correspondence: alexandra.papavlu@inflpr.ro or alexandrapalla@yahoo.co.uk

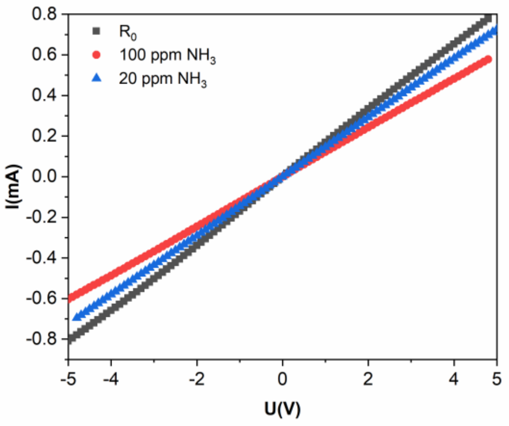

Figure S1. I-V characteristics of the laser printed CNW based sensors prior to their exposure to ammonia and when exposed to 20 ppm and 100 ppm.

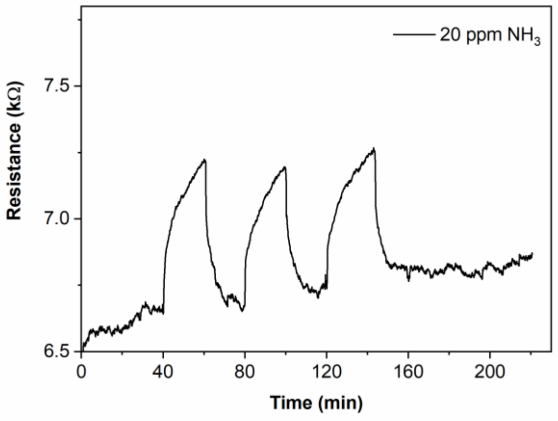

Figure S2. Laser printed CNW based sensor responses to successive 20 ppm NH_3 concentrations.
